# From cages to cage-free: A qualitative exploration of Chinese egg producers’ views on the opportunities and challenges to adopting cage-free egg production systems in China

**DOI:** 10.1017/awf.2025.10019

**Published:** 2025-07-10

**Authors:** Qing Yang, Fritha Langford, Belinda Vigors, Ruqian Zhao, Cathy M Dwyer

**Affiliations:** 1 Royal (Dick) School of Veterinary Studies, University of Edinburgh, Edinburgh, UK; 2 School of Natural and Environmental Science, Newcastle University, Newcastle upon Tyne, UK; 3 College of Veterinary Science, Nanjing Agricultural University, No. 1 Weigang Road, Nanjing 210095, China; 4 Department of Animal Behaviour and Welfare, Scotland’s Rural College (SRUC), Edinburgh, UK

**Keywords:** animal welfare, COM-B model, corporate sourcing, laying hen, market incentives, producer motivation

## Abstract

The transition from conventional cage systems to cage-free egg production in China remains limited despite apparently increasing consumer demand for cage-free eggs. This study interviewed 15 large-scale Chinese egg producers using cages and/or cage-free systems (i.e. single-, multi-tier and free-range) to investigate the perceived challenges and opportunities during the transition. The cage farms’ scales range from 110,000 to 30 million, while the cage-free farms keep between 12,000 and 300,000 laying hens. Drawing upon the COM-B model of the Behaviour Change Wheel, this study explored how producers’ Capabilities, Opportunities, and Motivations impact decision-making processes. Key findings reveal that cage and cage-free producers considered consumer demand and profitability as primary drivers for adopting cage-free systems. While free-range producers were more confident in the market, barn system producers faced greater uncertainty due to limited engagement from corporate buyers. Moreover, these cage-free producers believed reliable certification and labelling schemes to be critical for building consumer trust and ensuring the success of cage-free operations. All the participants perceived access to sufficient land and financial resources to be essential for a successful transition. While most studies propose education as a long-term strategy to promote the growth of the cage-free egg sector, our findings are the first to highlight that engaging corporate buyers and establishing trustworthy certification schemes are the most crucial short-term interventions required to drive the development of large-scale cage-free farms and support sustained improvements in animal welfare in China.

## Introduction

The adoption of cage-free housing systems in the egg industry is growing worldwide (Rodenburg. *et al.* 2020). Cage-free systems encompass both barn systems (i.e. single- or multi-tier aviaries) without outdoor access and free-range systems that provide outdoor space (de Luna *et al.* 2022). This transition from conventional cages is driven by scientific research and growing public concern regarding the welfare of farm animals (Hartcher *et al.* 2023; Rodenburg *et al.* 2020). While opinions as to what constitutes animal welfare may vary, most agree that it encompasses animals’ health, emotional state, and ability to perform natural behaviours (Fraser *et al.* 1997). Conventional cages offer benefits in terms of sanitation, disease management, and operational efficiency (Hartcher & Jones 2017). However, the restricted environment prevents hens from engaging in highly motivated behaviours, such as perching, nesting, foraging, and dust-bathing (Hartcher & Jones 2017). Furnished cages combine the production efficiency and hygiene advantages of conventional cages with some benefits of cage-free systems, such as enabling a wider range of behaviours. However, they still do not allow the full repertoire of natural behaviours, nor do they permit the full expression of certain key behaviours (Hartcher & Jones 2017). In contrast, although cage-free systems present certain challenges, such as keel-bone fractures, feather pecking, and smothering, hens in cage-free environments can display more natural behaviours and experience fewer health issues, including reduced osteoporosis and improved musculoskeletal health (Hartcher & Jones 2017). They may also experience less pain than those in conventional cages (Welfare Footprint Project 2022). Considering that the ability to express highly motivated behaviours is central to animal welfare, it is recognised that well-managed cage-free systems have the potential to improve animal welfare compared to conventional battery cages (Hartcher & Jones 2017). As such, in response to public concerns, some developed regions, such as the European Union (EU), have banned conventional cages (Rodenburg *et al.* 2020), and some countries have already banned furnished cages, with more planning to introduce similar bans in the near future (Marincheva & Manev 2023). Moreover, numerous food companies have committed to sourcing only cage-free eggs (Cage-Free Commitment 2024), with some, such as Unilever in Europe, successfully achieving their sourcing goals (Kollenda *et al.* 2020).

Despite these advancements in the EU (Appleby 2003; Balsiger 2016; Scrinis *et al.* 2017), the adoption of cage-free egg production in Asia, including China, is limited (de Luna *et al.* 2022; Rodenburg *et al.* 2020). Unlike the EU, China lacks regulations governing laying hen housing systems, and conventional cages dominate egg production (Yang 2021; Yang et al. 2018). In 2021, China raised around 3 billion laying hens (FAOSTAT 2022), with approximately 9% of eggs produced in free-range systems and 1% in barn systems (Yang *et al.* 2018). Although many corporations in China have pledged to source cage-free eggs (Cage-Free Commitment 2024), progress has been slow, with only a limited number fulfilling their commitments (Chicken Watch 2024). The slow process of sourcing cage-free eggs is likely tied to the low production of cage-free eggs in China (Global Coalition for Animal Welfare 2019), with only 10% of total egg production attributed to cage-free systems (Yang *et al.* 2018). Therefore, understanding the underlying reasons for this slow rate of change among Chinese producers is crucial for enhancing the welfare of millions of laying hens and developing effective strategies to accelerate this shift in the housing system.

Previous research on understanding the decisions of egg producers transitioning to cage-free systems has primarily been conducted in Europe and the United States (Caputo *et al.* 2023; Stadig *et al.* 2016). In the EU, regulations prohibiting conventional cages primarily drive producers to seek alternative housing systems (Stadig *et al.* 2016; Tolimir *et al.* 2020) and are often coupled with consumer demand and price premiums (Caputo *et al.* 2023; Stadig *et al.* 2016; Tuyttens et al. 2011). Additional factors also influence the decision-making process, including producers’ ability to manage cage-free farms (Caputo *et al.* 2023; Rodenburg *et al.* 2020), access to necessary resources (e.g. land, finances) (Stadig *et al.* 2016; Tuyttens *et al.* 2011), support from external stakeholders (e.g. government) (Caputo *et al.* 2023) and the availability of local knowledge on cage-free practices (de Luna *et al.* 2022; Hartcher *et al.* 2023). However, empirical evidence regarding the experiences and perceptions of producers in developing nations, such as China, remains scant (Bas Rodenburg *et al.* 2022).

In recent years, a small number of studies conducted in Asia, through interviews and surveys, explored the factors that affect Asian producers’ motivations to change to cage-free systems. In contrast to the findings in Europe, legislation is not found to be a major driver for transitioning to cage-free systems, as it is largely absent in the region (de Luna *et al.* 2022; Hartcher *et al.* 2023; Yang 2020). Moreover, for some producers, the current market demand in Asia is not strong enough to incentivise a switch to cage-free egg production systems (Hartcher *et al.* 2023; Yang 2020). While European producers require training in technical capacity building (Rodenburg *et al.* 2020), Asian producers need to strengthen their technical skills and develop knowledge of large-scale commercial cage-free production systems, as they are more familiar with smaller-scale free-range production systems (de Luna *et al.* 2022). As a result, the tailored strategies and recommendations proposed to encourage the adoption of cage-free egg production in Asia (de Luna *et al.* 2022) differ somewhat from those suggested solutions in a European context (Rodenburg *et al.* 2020). Therefore, given that the factors impacting producers’ behaviours in changing to cage-free systems are multifaceted and are affected by varied social, economic, and cultural contexts, it is essential to investigate Chinese producers’ resource-based, management-based, and farm-specific conditions (Chen *et al.* 2023), rather than applying experiences from other countries to China (Chen *et al.* 2023; Sinclair *et al.* 2022; Yang *et al.* 2024).

This study is the first to use the COM-B Model (Michie *et al.* 2014) to examine the social-environmental contexts of Chinese egg producers’ behaviours to devise support that enables producers to accelerate the transition to cage-free systems. Michie *et al.* (2014) suggest that human behaviour (B) can be understood as a result of Capability (C), Opportunity (O) and Motivation (M). These three interacting elements, as defined in Table 1, may help explain producers’ decisions regarding adopting cage-free systems in a China-specific context.

**Table 1.** Factors that impact egg producers’ decisions to adopt cage-free systems based on Michie *et al.* (2014): Capacity (Physical and Psychological), Opportunity (Social and Environmental), Motivation (Automatic and Reflective)

	Domain	Examples
Capacity	Physical	The physical strength, stamina, and motor skills required to adopt and operate cage-free systems
	Psychological	The mental processes, understanding, knowledge, and cognitive skills needed to adopt and operate cage-free systems
Opportunity	Social	Interpersonal influences, social cues, and cultural norms that either support or hinder participants from adopting cage-free systems
	Environmental	External elements such as time, location, and human and financial resources that facilitate or impede producers’ adoption of cage-free systems
Motivation	Automatic	Emotional reactions, desires (wants and needs), and impulses when participants consider adopting cage-free systems
	Reflective	Self-conscious intentions (i.e. plans) and evaluations (beliefs about what is good and bad) in participants’ decisions on using cage-free systems

Given the limited understanding of Chinese producers’ decision-making processes in the transition to cage-free systems, it is crucial to investigate their Motivations and different Capabilities and Opportunities. Arguably, failing to understand Capability, Opportunity and Motivation within an individual’s environment limits the potential effectiveness of interventional strategies, as they need to address wide-ranging influences on behaviour. Although the COM-B model has not previously been applied to producers’ decision-making processes on laying hen housing systems in the extant literature, this framework has been used to understand livestock stakeholders’ behaviours in other contexts, such as antibiotic use on Irish dairy farms (Farrell *et al.* 2023), sheep lameness management in Australia (Clark et al. 2024) and disease prevention on Dutch dairy farms (Jorritsma *et al.* 2023). In these studies, the identified factors influencing the targeted group’s capability, opportunity, and motivation to improve on-farm practices formed the basis for practical recommendations, and the COM-B model has been demonstrated as being applicable in various cultural contexts. By shedding light on the factors influencing producers’ adoption of cage-free systems, our research aims to provide insights that may inform targeted strategies to facilitate the transition toward cage-free egg production, thereby contributing to improving animal welfare in China.

## Materials and methods

### Interviews

This research was situated within a social constructionist paradigm, meaning that the researchers considered the social world not as an objective reality but as a collection of diverse interpretations offered by various individuals (Offermans & Glasbergen 2017). As such, the researchers strive to understand participants’ subjective experiences and how they make sense of their decisions rather than developing a generalisable understanding of a large group of people. In-depth, semi-structured interviews were selected as a suitable method because they enable researchers to define the areas to be explored with the flexibility to ask follow-up questions to pursue an idea or response in more detail (Gill *et al.* 2008). Moreover, this approach enables the discovery or elaboration of information critical to the participants but may not have been considered pertinent by the researchers (Gill *et al.* 2008). Semi-structured interviews have been used to understand producers’ perspectives, effectively revealing the factors that influenced egg producers’ decisions (not) to transition from cages to cage-free systems (Caputo *et al.* 2023; Yang 2020; Yang *et al.* 2024).

Drawing on the COM-B model, the interview guide was designed based on questions related to Capacity, Opportunity, and Motivation. Interview questions were designed to explore experiences related to both production systems and interviewees who used dual production systems were targeted. The interview guidelines were initially formulated in English to accommodate the diverse linguistic backgrounds of the authors, ensuring their collective input. Subsequently, the first author (QY) translated the guidelines into Mandarin Chinese to facilitate their application during the interview process. This linguistic duality helped maintain consistency in the research process while ensuring accessibility and comprehension for all authors. The interview questions began by understanding the participants’ experience using production system(s) and then focused upon their perceived driving and obstructive factors for transitioning to or maintaining cage-free systems. Additionally, producers’ perceptions of animal welfare and its importance in egg production and their impressions of laying hens were also asked. The interview guides were modified for cage egg producers and cage-free producers to make the questions more pertinent to their production experiences, as shown in Tables 2 and 3.

**Table 2.** Questions asked during interviews with Chinese cage egg producers (n = 4) to explore the factors influencing their decisions to adopt cage-free systems

Which province is your farm in?
What is the farm size (number of birds)?
What is your job position on the farm?
What production system do you use?
What led you to choose this production system?
What are the benefits of using this production system?
What are the disadvantages of using this production system?
How do you feel about using this system?
Have you heard of cage-free systems?
What do you think cage-free systems are?
What do you think of cage-free systems compared to cage systems?
What do you think are the reasons some producers choose cage-free systems?
If you were to use cage-free systems, what would you consider before you start?
Do you need support to change to cage-free systems?
What kind of help do you need to change to cage-free systems?
What can encourage more cage producers to use cage-free systems?
How likely will you adopt cage-free systems in the next five years?
What do you think of hens?
What do you think animal welfare is?
What impact do cages have on animal welfare?
What impact do cage-free systems have on animal welfare?
How important do you think animal welfare is in farming?

**Table 3.** Questions asked during interviews with Chinese cage-free egg producers (n = 11) to explore the factors influencing their decisions to adopt cage-free systems

Which province is your farm in?
What is the farm size (number of birds)?
What is your job position on the farm?
What production system do you use?
Did you use this production system since you set up this farm?
What led you to choose/change to this production system?
What are the benefits of using this production system?
What are the disadvantages of using this production system?
How do you feel about using this system?
What were your biggest difficulties when running your farm?
How did you overcome those challenges?
Do you need support in your farm operation?
What kind of support?
Who can provide such support?
Have you heard of cage-free systems?
What do you think cage-free systems are?
Have you observed any cage farms that want to try cage-free systems?
What are your suggestions for those producers who want to try cage-free systems?
What would help those producers change to cage-free systems?
What can encourage more producers to try cage-free systems?
Will you still run your farm in the next five years? How likely are you going to expand it?
What do you think of hens?
What do you think animal welfare is?
What impact do cages have on animal welfare?
What impact do cage-free systems have on animal welfare?
How important do you think animal welfare is in farming?

### Ethical considerations

Ethical approval for the interviews was granted by the Human Ethical Review Committee of the Royal (Dick) School of Veterinary Studies at the University of Edinburgh (code HERC_655_21). Prior to interviews being conducted, all respondents provided informed consent to participate, were informed that the data would be used solely for academic purposes, and were made aware that they could withdraw from the study at any time without any consequences.

### Participant recruitment

Purposive and snowball sampling techniques were used to recruit the participating producers. The recruited interviewees met the following criteria:

1. Business leaders from medium- to large-scale commercial laying hen farms in mainland China, as they were in the decision-making positions for selecting housing systems;
2. Egg farms with a minimum of 50,000 birds and 5,000 birds in annual stock for cage and cage-free egg farms, respectively;
3. The farms were located in one or more of China’s top egg-production regions: Shandong, Hebei, Henan, Liaoning, Jiangsu, Sichuan, Anhui, Hubei, Heilongjiang, and Jilin (Yang *et al.* 2018); and
4. The participants used cages (conventional cages) and cage-free systems, including single-level, multi-tier, and free-range systems.

Given that some poultry farms had been transitioning from cages to cage-free systems, producers using cages and cage-free systems concurrently were also included in the sample.

Fifteen egg producers (n = 15) were interviewed in the study. Participants were recruited through WeChat (Tencent Holdings Limited, Shenzhen, China), a widely used Chinese social media platform comparable to WhatsApp, and referrals from existing participants and industry associates. Recruitment procedures were terminated in accordance with the saturation criterion (i.e. no new information emerged) proposed by Braun *et al.* (2018). The professional roles of the participants included farm owners, managers, or senior leadership responsible for overseeing one or multiple farms. Among all the participants, four producers used only conventional cages on their farms, six used cage-free systems only, and five produced eggs in both cage and cage-free systems. For the sake of simplicity, all participants are collectively referred to as ‘producers.’ Detailed information regarding the farms’ locations, scales, and housing systems is available in Table 4.

**Table 4.** Geographic locations, estimated annual flock sizes, and egg production systems used by farms or companies the interviewed participants (n = 15) represent

Participant Number	Locations	Farm scales	Production systems
1	Sichuan	1 million	Conventional cages
2	Nationwide	13 million	Conventional cages
3	Hebei	12,000	Free-range
4	Shanxi	Cage: 350,000 Cage-Free (Multi-tier):40,800	Conventional cages Cage-Free (Multi-tier)
5	Sichuan	20,000	Free-range
6	Nationwide	Cage: 110,000 Cage-Free (Multi-tier): 25,000	Conventional cages Cage-Free (Multi-tier)
7	Nationwide	Cage: 300,000 Cage-Free (Single-tier): 10,000	Conventional cages Cage-Free (Single-tier)
8	Henan	500,000	Conventional cages
9	Guangdong	1.2 million	Conventional cages
10	Nationwide	300,000	Free-range
11	Henan	40,000	Free-range
12	Beijing	100,000	Free-range
13	Nationwide	Cage:30 million Cage-Free (Multi-tier):200,000	Conventional cages Cage-Free (Multi-tier)
14	Fujian	Cage: 1 million Cage-Free (Single tier): 100,000	Conventional cages Cage-Free (Single-tier)
15	Nationwide	100,000	Cage-Free (Single-tier)

### Data collection

Fifteen semi-structured interviews were conducted via telephone in Mandarin Chinese between March and July 2021 with interviewers following the interview guides during questioning. Further in-depth inquiries were conducted to gain insight into participants’ experiences with cage and cage-free systems. Participants’ responses primarily guided the discussions, encouraging open and comprehensive dialogue. All interviews were recorded using a Mi 10 mobile phone from Xiaomi Communication and Technology Ltd, China, and subsequently transcribed into written Mandarin Chinese by QY. The transcriptions were then uploaded and analysed using Nvivo (QSR International Pty Ltd 2018).

### Data analysis

Template analysis was applied to analyse all the data. As a form of thematic analysis, template analysis uses hierarchical coding, which allows a structured and iterative approach with flexibility throughout the coding process (King 2012) and helps understand a relatively under-explored topic (Smid *et al.* 2021). Due to the many subthemes inherent in our relatively unexplored research questions, template analysis was a well-suited approach for organising and analysing our data. Template analysis was employed in previous studies to analyse qualitative data on farmers’ perceptions and understanding of livestock production, animal welfare and husbandry practices in China (Chen *et al.* 2021; Chen & Weary 2022).

QY followed the recommended procedures for the template analysis application (King & Brooks 2017) and also listened to all recordings, verifying them against the transcribed manuscript. Several themes and subthemes were defined *a priori*, which corresponded to the components of the COM-B framework. Two primary themes (i.e. facilitators and barriers to adopting and sustaining cage-free systems) and six secondary themes (i.e. physical/psychological capabilities, physical/social opportunities and reflective/automatic motivations) were defined prior to the preliminary coding. Five interviews were then selected, and QY coded a text segment with a phrase under a secondary theme. For instance, the codes ‘unavailability of land’ and ‘unavailability of money’ were categorised within the secondary theme of ‘physical opportunities’ under the primary theme of ‘barriers to transitioning to cage-free.’ QY used the list of codes and themes drawn from the five interviews as a template and applied it to the remaining ten transcripts. Codes and subthemes were refined and divided during the process, and new codes were added. An audit trail was also maintained, which included reflective comments on coding choices and the reasons behind particular modifications.

Participant quotes are used to substantiate the identified themes and represent the key findings of this study. The quotes were selected based on their succinct encapsulation of a theme, representation of participants’ views, and ability to highlight differences among participants. The selected quotes were translated from Mandarin Chinese to English by QY. This singular translator approach was adopted to enhance the reliability of translation and interpretation (Twinn 1997). Additionally, a bilingual researcher with expertise in a related research discipline cross-checked some quotes to preserve the intended meaning and context in Mandarin Chinese. In included quotations, square brackets were utilised to incorporate contextual information, ensuring the readers’ clarity. Since this paper focuses on the opportunities and barriers for Chinese egg producers to transition to and maintain cage-free systems, findings regarding participants’ perceptions of and attitudes toward animal welfare are reported in a different paper (Yang *et al.* 2024).

## Results

**Figure 1.**
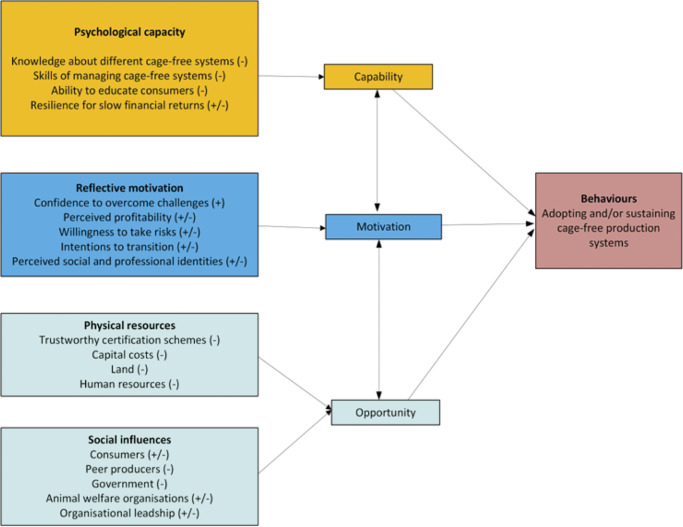
Capacity-, opportunity-, and motivation-related factors identified by all interviewed participants (n = 15) that influenced decisions to adopt or not adopt cage-free egg production systems.

Eighteen themes were identified and mapped to the COM-B elements. Figure 1 illustrates the factors that influence participants’ decisions regarding egg production systems. Facilitators for transitioning to cage-free egg production are marked with a positive symbol (+) to indicate favourable factors, and barriers are represented by a negative symbol (–) to denote challenges or constraints.

### Producers’ capability to adopt cage-free systems

Themes related to physical capacity were not identified. The findings reveal psychological capability barriers to transitioning to cage-free systems, including inadequate knowledge of different cage-free systems, insufficient cage-free system management skills, inabilities in consumer education, and their resilience in achieving financial returns.

### Producers’ inadequate knowledge of cage-free systems

While most participants were familiar with the term ‘cage-free’, some lacked a comprehensive understanding of the various systems it encompasses. Most cage producers equated cage-free systems with single-level or free-range systems, indicating that they were unfamiliar with aviary or multi-tier systems as types of cage-free production systems. One participant stated: “*My understanding of* [cage-free] *is free-range; it is either housing the birds indoors or free-range*” (P12). Another participant added, “*I know more about the free-range systems with outdoor access… I know very little about the modern cage-free systems… I only read about it in books and saw it in online videos*” (P1). Aviary systems were perceived as uncommon in China, where cages remain the mainstream production method. A participant noted, “*I haven’t heard of* [aviary systems] *in commercial egg production… most use cages*” (P8). This lack of knowledge created psychological barriers to adopting cage-free systems beyond free-range and single-level systems.

### Producers’ insufficient skills in managing cage-free systems

Some cage producers suggested that insufficient technical capacities in managing cage-free systems, particularly in disease control and vaccination, posed challenges to adopting cage-free systems. Producers’ technical capabilities involve learning and applying new management techniques, which are a cognitive process, not physical strength and endurance (e.g. manually vaccinating birds). One participant explained, “*It is inherently harder to manage cage-free systems… In China, disease control requires vaccinations, and administering vaccines poses a significant challenge* [in cage-free systems]” (P1). Another added, “*The Chinese circumstance is completely different from that of Europe and the United States. Our disease control challenge is greater than Europe and America’s, so their experience cannot be directly applied*” (P13). As such, it is essential to develop localised technical knowledge and standards. For example, one stated, “*We are using imported facilities and equipment, and the manufacturer provides some support. However, their support may not be useful… because the climate and the breeds raised here are different. Various production data, including ventilation and air exchange designs, are incompatible with local conditions here*” (P6).

### Producers’ inability to educate consumers

Participants also faced difficulties in educating consumers regarding animal welfare and cage-free products. Many consumers were unaware of animal welfare and the differences between caged and cage-free eggs. One participant explained, “*When you mention welfare farming, nine out of ten people may ask, ‘What is welfare farming?’ The high educational cost of the market is a significant difficulty for small and micro-enterprises like us; it’s a critical issue*” (P6). Another added, “*From the perspective of the farms, it’s not something we can drive…we cannot push* [the transition to cage-free production] *…; we neither have the willingness nor the capability to do so*” (P7). As such, cage-free producers sought multiple stakeholders to take part in “educating the market”:

“*Consumers have very limited awareness of animal welfare. The promotion efforts we undertake are likely to have minimal impact. We need the involvement of the government, authorities, media, producers, and retailers to pay attention to the consumer awareness process and to communicate more relevant information…Our influence* [on consumers’ awareness] *is very weak, extremely weak*” (P11).

### Producers’ resilience for slow financial returns

Participants’ discussion also revealed that some cage-free producers have demonstrated resilience in the face of slow financial returns. Such capacity for resilience may help sustain cage-free operations: “*Economic viability is the fundamental basis for a business’s survival… However, it can be gradually achieved over time. It’s important not to expect all profits to be realised in one go*” (P11). Consequently, a few cage-free producers who have been engaged in cage-free production for over a decade confirmed the necessity of being patient: “*I do recognise* [the meaningfulness of] *this industry because this industry has a future…*[but the financial returns in] *agriculture are indeed very slow*” (P5); and “*…we can only wait for the market to grow…we have to wait patiently…the maturity of our entire market takes time*” (P14).

In contrast, for cage producers, the barrier lies in the pursuit of immediate profitability, a perspective grounded in the belief that it is imperative for the survival of their companies. “*We have always chosen high-density cage systems because they allow us to produce the most eggs with the minimum investment cost. Only under these conditions can we barely sustain our survival*” (P13), and “*The profits from caged production are higher than cage-free production… However, it is still challenging for caged farms to maintain high profits. Thus, we will not consider exploring or trying new, less common systems like cage-free*” (P9).

In summary, limited knowledge, technical capabilities, and difficulties in consumer education impede producers from switching to cage-free systems. Cage-free producers’ resilience in pursuing long-term financial returns facilitates their commitment to continuing cage-free production.

### Producers’ opportunity to transition to cage-free systems

Producers’ decisions to transition to cage-free systems were shaped by social influences from stakeholders (i.e. consumers, peer producers, government, animal welfare organisations, and company leadership) and the availability of physical resources, including certification schemes, capital, land, and human resources.

### Social influences on changing to cage-free egg production

Consumers were identified as the primary stakeholders influencing producers’ decisions. Nearly all participants agreed that their choices of production systems depend on consumers’ purchasing behaviours: “*…when consumers make a choice* [of buying cage-free products]*, it determines producers’ choices of cage-free systems*” (P1). Most free-range producers were motivated by established consumer demand in regional markets. One explained that local preferences for higher-quality eggs and meat drove their decision to start free-range farms: “*Chicken in free-range systems can move around, and the meat tastes firmer, which is more suitable for the consumer’s preference in the local region*” (P5). One cage-free producer using an aviary system admitted he wanted to supply his corporate client who had pledged to source cage-free eggs and advised him to start the operation: “*I started the cage-free farm because the large corporations want to implement their cage-free commitment and I want to try it and meet their demand*” (P4). Nevertheless, some participants were sceptical of consumer demand and described consumers’ willingness to pay as insufficient. One explained, “*The demand is not strong in China at present, and the price consumers are willing to pay for cage-free eggs is not very good either*” (P15). Another noted, “*There is market demand for cage-free eggs, but the number of consumers willing to pay is small*” (P1).

Some cage-free producers perceived peer producers as a barrier to sustaining cage-free operations. They expressed frustration with those peers’ deceptive practices, who falsely labelled caged eggs as cage-free. One participant explained, “*Consumers are deceived because their eggs are not truly cage-free eggs but rather caged eggs, but there is no inspection or integrity, so the* [cage-free] *market is disrupted*” (P14). Another free-range producer emphasised that “*no one is going to buy more expensive eggs, and they can’t differentiate between caged eggs and free-range eggs …* [without effective regulation]*, it is hard to control* [mislabeling]” (P3).

When asked who should regulate the cage-free market, government involvement was seen as limited but necessary for the long-term growth of the cage-free sector. Participants noted that the government prioritised food supply over higher-welfare products, which most consumers could not afford. One participant explained, “*Currently, the government is the best authority to regulate cage-free certifications and labelling…but the government is not motivated to get involved* [in the issue] *at present*” (P1). Another added, *“The government in China would first solve the issue of egg supply for consumers and then consider the issue of eating better*” (P2).

Moreover, although animal welfare groups were perceived as an important driving force in promoting cage-free production, some producers indicated that these groups could enhance their efforts to raise awareness about cage-free products, implying that the pressure exerted by these organisations was not uniformly perceived as robust. For instance, a cage producer remarked, “*To promote the concept* [of cage-free eggs]*, some civil organisations, in the future but not now, can promote it through the perspective of animal rights, it might create a driving force*” (P1). Another cage-free producer also proposed that international animal welfare organisations could provide and regulate the certification and labelling systems but noted their limited recognition among consumers: “*The consumers do not necessarily recognise it if I am certified by an international certification organisation… because the consumers never heard of the organisation and do not recognise its credibility*” (P3).

Participants also highlighted the importance of leadership within production companies. Decisions to adopt cage-free systems were often driven by company leaders seeking to produce safer, higher-quality products. One participant explained, *“The owner had the initial idea of producing agricultural products that were more ecological, natural, and high-end. He wanted to produce products of better safety and quality… so he built this farm*” (P12).

### Resources to facilitate the transition to and sustainment of cage-free egg production

The interviews identified several key barriers to transitioning to and sustaining cage-free egg production. A significant challenge was the absence of reliable certification schemes, which undermined consumer trust and discouraged corporate buyers. One producer highlighted how this absence discourages retailers and businesses from purchasing cage-free eggs: “*At present, I think the retailers won’t accept cage-free eggs because there are no standards…how can you prove these are cage-free eggs? You can’t take all the clients to visit the farm to prove it*” (P4).

Land constraints were another major obstacle. Cage-free systems require more space than conventional cage systems, but China’s land policies prioritise arable land for crop production, making it difficult to secure adequate space for animal farming. One participant stated, “*There are constraints on land sourcing in China, and a cage-free farm requires a larger area… Finding suitable land is difficult*” (P4).

Financial barriers also prevent producers from expanding cage-free operations. Many participants mentioned the high capital costs of infrastructure upgrades as challenging. One producer summarised the difficulty: “*If you intend to expand the cage-free farms with available funds, the likelihood is almost zero. This is why I won’t transition to large-scale operations in the short term — it’s simply not financially feasible*” (P6).

Additionally, there is a shortage of skilled human resources, particularly managers and advisors trained in cage-free systems: “*Cage-free production can be unstable, and the management team don’t have much experience, so they have to be trained from scratch. There are no experienced managers to hire*” (P6). While it is easier for free-range producers to find technical advisors, it is hard for aviary producers to access them due to their recent adoption in China. One producer stated, “*Currently, no one can provide advice on* [managing aviary systems]*. Everyone is still relying on exploration and experimentation*” (P13).

In summary, consumer demand for cage-free products and supportive leadership within the company enable the adoption and sustainment of cage-free systems. However, significant barriers remain, including hesitant buyers, limited engagement from government and animal welfare organisations, peer producers employing misleading practices, and critical resource shortages, such as reliable certification schemes, adequate funding, sufficient land, and skilled human resources.

### Producers’ motivation to adopt and maintain cage-free operations

No themes related to automatic motivations, such as impulses and reflex responses, were found. However, five themes highlighted the participants’ reflective motivation, including their confidence in overcoming operational challenges on cage-free farms, obtaining profitability, willingness to take risks, the perceived social and professional identities, and intentions to transition to cage-free systems.

### Producers’ confidence to overcome challenges during transition

Cage-free producers demonstrated strong confidence and a ‘can-do’ attitude in overcoming challenges, which enabled them to develop and sustain their operations. For example, some sought financing solutions to address funding issues, such as obtaining loans and negotiating egg prices with buyers:

“*We are currently in the process of obtaining some financing. Alternatively, we might consider… negotiating the price of eggs with a buyer company and offering them a discount* [to sell more eggs]*. These are all helpful* [ways to address funding issues]” (P4).

Additionally, all cage-free producers emphasised the importance of a learning mindset to build the skills to manage cage-free systems. For example, one free-range producer highlighted the value of experience in avoiding mistakes:

“*After accumulating experience over many years, including … improving procedures in vaccine administration.* [if we can ensure that] *preventive measures are well-implemented, major issues are less likely to occur*” (P5).

### Producers’ motives to obtain profitability

Obtaining price premiums and achieving profitability were identified as key drivers for adopting cage-free systems. Participants recognised the potential higher egg prices to make cage-free systems profitable. A producer using aviary systems stated, “*…in the future*, [cage-free eggs] *may have a good prospect…caged eggs might not be profitable, but the price of cage-free eggs will be higher and can be profitable… the price of cage-free eggs must be higher. Otherwise, nobody wants to do it*” (P4).

Another producer stated, “*With the improvement of living standards, some people have a demand for products of higher quality … it is a business, so the first consideration is profit … if it is profitable, one can do it*” (P10).

However, some large-scale cage producers viewed profitability as a barrier, expressing concerns about input-output ratios and weak market demand. One producer explained:

“*For us, it’s not a matter of capital investment; it’s about calculating the return on investment… specifically evaluating the costs and benefits, with a primary focus on the input-output ratio… The ratio between investment and returns is crucial, a key consideration for decision-making* [on starting cage-free production]” (P2).

Another large-scale egg company also explained why it abandoned its plan to start a cage-free farm: “*We wanted to start a cage-free farm. Initially, we considered housing millions of birds in aviary systems, but the financial projections failed to achieve our anticipated profitability*” (P13).

Meanwhile, some cage-free producers admitted they were struggling with maintaining profitability and expanding their cage-free operations due to uncertain demand:

“*The core issue lies in a confirmed demand from end consumers. The demands we currently see are very uncertain… and I cannot invest in such a high-cost farm for this uncertainty…Up to now, we haven’t sold many eggs… Most* [cage-free] *eggs are still sold* [at the price of] *caged eggs*” (P7).

### Producers’ willingness to take risks

Despite uncertainties in market demand and profitability, several large-scale, cage-free producers demonstrated a strong risk-taking mindset, which drove their decision to transition. One producer shared:

*“I don’t want to do something that lacks a sense of fulfilment. Pursuing only efficiency without considering spiritual satisfaction is something ordinary farmers can do and do well. Who will take on these challenges* [in running cage-free businesses] *except for people like us who are driven by more than just financial goals?*” (P6).

Conversely, cage producers were more risk-averse, preferring the certainty of market demand for cage eggs. One cage producer explained:

“*It’s about* [market] *certainty. More companies will adopt cage-free systems if there is a relatively high level of certainty and stronger market demand. Currently*, [we] *will not consider the less commonly used housing systems, which may involve more uncertainties and potential* [financial] *losses”* (P9).

### Producers’ intentions to transition to and maintain cage-free systems

Interestingly, all the cage-free producers expressed strong intentions to maintain and expand their operations despite the perceived barriers. All the participating free-range producers were confident they could supply their targeted market and maintain their free-range businesses. In contrast, participants who used cage-free systems without outdoor access were driven by the potential to meet future corporate demand for cage-free eggs, as one aviary producer explained:

“*As big companies have the policies* [of sourcing cage-free eggs]*, I want to try and see if cage-free production is feasible or not. Some companies have committed to it by 2025; perhaps, by that time, some big companies will start transitioning*” (P4).

While the corporate buyers’ timelines and willingness to pay were unclear, participants’ motivation for supplying these corporates was primarily associated with their mentality of ‘seizing the market initiative’ at an early stage. One cage-free producer’s farm is not profitable, but he still had business expansion plans. He explained it was important to start early to capture a market opportunity:

“*We saw the potential market demand. We talked about the 2025 commitment…If consumers show a certain market demand by then, the business principle remains the same: whoever can first meet that demand will be able to capture that segment of the market. It’s a fundamental business principle…this is a key consideration*” (P7).

However, some cage producers, mostly unaware of corporates’ cage-free egg commitments, expressed no intention to transition and doubts regarding future demand and consumer awareness. One producer explained:

“*I don’t think I will* [change to cage-free production]*, unless market demand changes greatly, or consumer awareness improves, but I believe they are unlikely to change in five years*” (P1).

### Producers’ perceived social and professional identities

Cage-free producers viewed their systems as better aligned with social responsibility, benefiting animal welfare and human well-being. One producer shared:

“*Engaging in ecological agriculture aligns with our initial intentions in agriculture and represents a social responsibility. Transitioning away from cage systems is not only about the welfare of animals but also about the welfare of humans. Producers should not prioritise short-term economic benefits but shoulder their social responsibilities*” (P11).

In contrast, caged producers emphasised the affordability and efficiency of cage systems in meeting food supply needs, particularly for low-income consumers. One producer argued:

“*China has a large population, and people come from different walks of life with different demands… people need caged and cage-free products. It’s unlikely that one can completely replace the other. Let’s work together and accommodate different demands*” (P5).

In conclusion, the key motivations for cage-free producers to transition, maintain, and expand their operations include confidence in overcoming challenges, potential for profitability, risk-taking attitudes, strong intentions to seize market opportunities, and alignment with their social and professional identities. The primary driver is the anticipated demand from food businesses with cage-free commitments by 2025. While uncertainty persists regarding corporate timelines and willingness to pay, cage-free producers are motivated to capture future market opportunities. Conversely, cage producers remain hesitant due to concerns about profitability, market uncertainties, and prioritising food affordability.

## Discussion

This study applied the COM-B model to examine the capabilities, opportunities, and motivations influencing Chinese egg producers’ adoption and sustainment of cage-free systems. Psychological capacity barriers included inadequate knowledge of cage-free systems, insufficient technical capacities, and inability to educate consumers. Resilience in seeking long-term economic returns supports producers in maintaining their cage-free farms. Opportunities were shaped by social influences from various stakeholders, including consumers, peer producers, government, animal welfare organisations, and company leadership, as well as access to physical resources, such as certification schemes, financial capital, land, and skilled human resources. Reflective motivations were driven by producers’ confidence in overcoming challenges, pursuit of profitability, willingness to take risks, perceived social and professional identities, and intentions to transition. These findings highlight the complex interplay of factors shaping producers’ behaviours and offer insights to support the growth of cage-free production in China.

### Motivational drivers and barriers

The results confirm that profitability is the primary motivator for producers to adopt and maintain cage-free systems. Research in Asia has consistently shown that consumer-driven demand is the key driver of cage-free adoption in regions without legislative bans on conventional cages (Chen *et al.* 2023; de Luna *et al.* 2022; Hartcher *et al.* 2023). Particularly, free-range producers cater to established individual consumer demand in regional markets, whereas producers using barn systems target corporate buyers with cage-free commitments. This segmentation reflects the nuanced motivations which are also observed by Chen *et al.* (2024) in interviews with producers using different cage-free systems.

The stronger market presence of free-range eggs in China can be attributed to cultural and historical factors. Free-range egg production is a well-established farming and cultural practice (Yang 2021), and consumer demand for free-range products has been longstanding (Liao *et al.* 2023; Liu *et al.* 2023). Consumers associate free-range eggs with traditional values (e.g. indigenous chicken eggs), health benefits (e.g. nutrition) and altruism (e.g. protection of the environment and animals) and are willing to pay premium prices (Chen *et al.* 2024). In contrast, ‘cage-free’ remains a less-familiar concept (Chen *et al.* 2024), with many consumers reportedly purchasing only free-range eggs (The Poultry Site 2022). While marketing strategies, such as farm visits, have been effective for free-range producers in stimulating targeted consumers’ purchasing behaviours and building trust, the unfamiliarity presents challenges for barn system producers in using similar approaches to engage with individual consumers (Chen *et al.* 2024).

In contrast to the individual consumers’ willingness to pay for free-range eggs, corporate buyers with cage-free commitments often resist paying higher prices for cage-free eggs (Chen *et al.* 2024). Furthermore, a lack of regulatory pressure and the limited availability and traceability of cage-free eggs also hindered the progress of sourcing cage-free eggs in China (Global Coalition for Animal Welfare 2023). Therefore, despite optimistic projections of a 1.8 billion annual increase in demand due to corporate cage-free commitments (Food Navigator Asia 2022), the progress of sourcing cage-free eggs in Asia-Pacific lags behind other regions, with only 55% of conversions completed, compared to Europe and America with 80 and 72%, respectively (Egg Track 2024). The ‘soft commitments’ lead to uncertainty and risks in the supply chain, as producers can only sustain their cage-free operations once they have long-term commitments from their corporate buyers (Caputo *et al.* 2023). Additional research is needed to understand the incentives and challenges perceived by corporate buyers in China to bridge the pledge-buy gap and support producers’ cage-free operations.

This study suggests that cage-free producers who are confident in overcoming challenges, exhibit a risk-taking propensity, align with their professional identity, and have an intention to expand cage-free production are more likely to sustain their cage-free farms. Previous studies have found that producers’ self-confidence, risk-taking attitudes, perceived identity in farming practices, and intention play important roles in their decision-making processes for improving animal welfare (de Lauwere *et al.* 2012; Hansson & Lagerkvist 2014; Kauppinen *et al.* 2010; Vigors *et al.* 2023). Producers’ experience with cage and cage-free systems influences their perceptions and commitment. Those who have previously used a particular system tend to view it more positively (de Lauwere *et al.* 2012; Stadig *et al.* 2016; Tuyttens *et al.* 2011). Given these factors, as a short-term strategy, it might be more strategic to collaborate with cage-free producers who exhibit these motivational traits rather than engage with those who lack them, as they are more likely to sustain and expand cage-free production.

### Capability drivers and barriers

The findings show that the participating cage producers have limited knowledge of aviary systems and insufficient managing skills of cage-free systems, which is aligned with earlier studies in Asia reporting limited awareness of and experience with aviary systems among producers (de Luna *et al.* 2022; Hartcher *et al.* 2023). In contrast to cage producers, cage-free producers demonstrated better knowledge of various cage-free systems, and they were more confident in solving technical challenges when they admitted their technical capacities were lacking. As such, these cage-free producers were willing to continue cage-free production. These results are consistent with previous research showing that technical knowledge and perceived competence strongly influence producers’ willingness to adopt higher-welfare systems (de Lauwere *et al.* 2012). This knowledge discrepancy may reflect recent advancements in knowledge-sharing initiatives targeting the cage-free sector, such as the China Egg Quality and Hen Welfare Summit, which have facilitated knowledge dissemination among cage-free producers (FAI Farms 2023). Furthermore, with the growing adoption of aviary systems, these producers are accumulating experience in managing large-scale cage-free operations (FAI Farms 2022). This suggests that the next step should prioritise technical capacity building among existing cage-free producers to sustain the cage-free sector rather than raising awareness of the cage-free topic among cage producers who lack comparable experience and expertise.

This study highlights the importance of developing localised management practices and standards tailored to the Chinese context as a long-term strategy to grow the cage-free sector in China. Producers in the study emphasised challenges related to disease control, biosecurity, and equipment compatibility, mirroring findings from other studies in Asia (de Luna *et al.* 2022; Hartcher *et al.* 2023). Cage-free systems require a higher level of knowledge and management skill than conventional cages in areas such as nutrition, rearing and vaccination (Rodenburg *et al.* 2022). That said, these producers also expressed confidence in their ability to overcome these challenges through self-learning and practical experience, with some reporting significant progress in improving production performance as they gained expertise in managing cage-free systems. These findings suggest that technical capacity building, while important, serves more as a long-term strategy to drive the development of the cage-free sector in China rather than being a critical factor limiting the current operations of cage-free farms. Given the absence of animal welfare legislation in China, capacity-building initiatives are crucial to improving the competitiveness and sustainability of cage-free farms (de Luna *et al.* 2022). Evaluating the long-term effectiveness of educational initiatives, such as establishing cage-free model farms and training centres in China (The Poultry Site 2023) presents an avenue for future research.

Some cage-free producers reported being more economically resilient than their caged counterparts, partly because they recognise the limited consumer understanding of cage-free systems. They understand that achieving financial returns from cage-free production is gradual, requiring time to educate the market and overcome consumer misconceptions. The limited awareness of animal welfare and cage-free eggs among the large Chinese consumer population, compounded by a preference for free-range products (Chen *et al.* 2023), creates further barriers for barn producers. As a result, producers using barn systems may face greater difficulties in maintaining economically viable cage-free farms. Financial strategies, such as equitable subsidy programmes, could help these producers overcome these challenges.

### Opportunity driver: Physical resources and social support

Cage-free producers expressed that a lack of certification schemes impeded producers’ development of cage-free businesses. In China, where government regulations for animal welfare standards or labelling are absent, private certification and labelling schemes have emerged to assure consumers and foster trust in the Chinese cage-free egg market (Scrinis *et al.* 2017). When the interviews occurred, two industry-initiated, cage-free certification standards were implemented to certify cage-free eggs, and dozens of farms had been certified (Animal Welfare 2024). However, most producers in the current study were unaware of these certification schemes or complained about the untrustworthiness of certifications and a lack of labelling transparency. This is inconsistent with the findings of Chen *et al.* (2024), which showed that most interviewed egg producers were certified and used the certification schemes to communicate with individual and business buyers about the authenticity of cage-free eggs. The inconsistency might be because the interviews in this study were conducted 1–2 years earlier (March to July 2021) than those in the study of Chen *et al.* (2024) (August 2022 to September 2023). Hence, producers’ knowledge and information regarding cage-free certification and labelling in this study was still limited. Further research is needed to improve recognition of certification and labelling schemes among producers and buyers in China.

The transition to cage-free systems is also heavily influenced by access to other resources, particularly land, financial capital and experienced cage-free workers and advisors. Producers with adequate resources have a greater likelihood of succeeding compared to those without who face significant barriers. Land scarcity, linked to China’s prioritisation of arable land for crop production (Huang 2023), has been consistently identified as a major obstacle (de Luna *et al.* 2022; Yang 2020). Similarly, the high capital costs associated with cage-free infrastructure limit the feasibility of large-scale transitions (Caputo *et al.* 2023; de Luna *et al.* 2022; Tuyttens *et al.* 2011), especially for free-range systems, where costs can be up to 70% higher than those of conventional set-ups (Sumner *et al.* 2011). Moreover, a lack of knowledgeable human resources regarding aviary systems may be due to their limited application and recent adoption compared to free-range systems (Chen *et al.* 2023). It also takes time for management knowledge and expertise to develop (Schuck-Paim *et al.* 2021). Therefore, a more strategic focus on resource-rich producers could accelerate more effective growth of cage-free production compared to attempting to bring onboard under-resourced producers (Caputo *et al.* 2023; Rodenburg *et al.* 2022).

Participants in this study pointed out that consumers, peer producers, government, animal welfare organisations and company leadership can shape the development of cage-free egg production. As discussed earlier, consumer demand and purchasing behaviours affect producers’ decisions to start and continue cage-free businesses. For the pursuit of profitability, cage producers labelled cage eggs as ‘cage-free’, which impeded the willingness of cage-free producers to develop their cage-free operations. Without government-enforced standards for farm animal welfare or labelling, egg producers may employ misleading terms, such as ‘rustic eggs’ (tu jidan土鸡蛋), ‘grass eggs’ (cao jidan 草鸡蛋), ‘agrestic eggs’ (ben jidan 笨鸡蛋) and ‘wood eggs’ (chai jidan 柴鸡蛋). These terms evoke a natural, idyllic setting, often associated with traditional farming techniques that prioritise quality over quantity. However, such claims are not monitored for authenticity, and scepticism restrains corporations and consumers from purchasing cage-free eggs (The Poultry Site 2022).

Meanwhile, although some animal welfare organisations play a role in promoting cage-free egg production, the number of these farm animal welfare groups is small, with less attention paid to farm animal welfare than other animal welfare topics, such as wildlife (Carnovale *et al.* 2021), their impact on the public may be limited. As such, although producers do not perceive government involvement as an immediate or critical factor in changing the *status quo*, it remains a necessary long-term strategy for developing the cage-free sector. Future governmental actions could include setting welfare standards, regulating certification and labelling, and addressing systemic challenges such as consumer affordability of higher-welfare products. Lastly, engaging resourceful cage-free producers with supportive leadership may represent a strategic approach to expanding the cage-free sector. These producers are better positioned to overcome barriers and serve as role models for those who want to transition to cage-free systems.

Since the transition to cage-free systems is a topical issue in farm animal welfare in China, and the authors were known to some participants as animal welfare advocates, participants’ interview responses may be subject to social desirability bias. Bearing this potential limitation in mind, the interviewer adopted mitigation strategies suggested by Bergen & Labonté (2020) to reduce the bias, including indirect questioning techniques, explaining the study’s purpose and confidentiality measures, ensuring participants trusted their anonymity, framing questions to acknowledge challenges, and using follow-up questions to clarify vague responses. The results did not reveal strong indications of social desirability bias. Participants gave balanced responses, discussing both the advantages and disadvantages of cage and cage-free housing systems. Many cage producers openly discussed barriers such as profitability and market demand, with some explicitly stating they had no intention of transitioning to cage-free systems. Indicators of bias, such as vague answers, denial of challenges, excessive praise for cage-free systems, or inconsistent responses (Bergen & Labonté 2020), were not evident. While social desirability bias cannot be excluded entirely, the strategies employed and the transparency of responses suggest it had limited influence on the findings.

### Animal welfare implications

Cage-free egg production systems are promoted in China as alternatives with higher animal welfare potential than conventional cages; however, the transition to cage-free production is slow. This study aims to understand the factors influencing Chinese egg producers’ decisions regarding housing systems, which could help identify strategies to address concerns about switching to cage-free production. The findings suggest that cage-free producers, particularly those operating large-scale, indoor systems with sufficient resources, are likely to commit to and sustain their cage-free operations in the short term. For these producers, a critical priority is establishing relationships with corporate buyers to ensure the steady sourcing of cage-free eggs, allowing their farms to thrive. In the long run, external technical and social support from various stakeholders can contribute to the growth of the cage-free egg sector. Further research is needed to develop marketing opportunities for cage-free producers, focusing on the challenges and opportunities perceived by corporate buyers in sourcing cage-free eggs in China. By understanding the purchasing behaviours of corporate buyers, cage-free operations can be sustained and expanded, ultimately improving animal welfare by freeing more laying hens from conventional cages.

## Conclusion

This study sheds light upon the complex challenges and opportunities faced by Chinese egg producers as they transition to cage-free systems. The findings suggest that compared to less-motivated cage producers, those already producing cage-free eggs are more willing and committed to obtaining the necessary skills and resources to sustain their operations. Additionally, producers using barn systems without outdoor access, compared to those using free-range systems, need more support to maintain their large-scale cage-free farms. For these producers, encouraging corporate buyers to source cage-free eggs and establishing trusted certification schemes are essential for sustaining and expanding the cage-free sector in the short term. In the long term, addressing technical and operational challenges through targeted capacity-building efforts and fostering collaboration among stakeholders will be key to maintaining cage-free production. By focusing on well-resourced producers and strengthening their engagement, the cage-free sector in China can not only grow but also help improve animal welfare standards across the industry. Further research is needed to examine the incentives and challenges corporate buyers face in sourcing cage-free eggs, as well as the acceptance of certification schemes in the Chinese market. Understanding these factors will help support a more effective transition and contribute to a more sustainable and welfare-friendly egg production system in China.

To the authors’ knowledge, this study represents the first application of the COM-B model to understand the decision-making processes of Chinese egg producers as regards the adoption and maintenance of cage-free egg production systems. The study aims to investigate participants’ perspectives, views, and beliefs regarding the transition to cage-free egg production systems rather than providing a comprehensive overview of the entire population. Furthermore, the findings highlight the usefulness and applicability of the COM-B model in understanding the factors influencing the transition of the egg industry to cage-free housing systems. Applying the COM-B model enabled the authors to better structure the interview questions and analysis, facilitating a nuanced examination of Chinese producers’ complex decision-making processes. This approach provided valuable insights into designing targeted interventions and policies. Based on an understanding of producers’ behaviours, future research can further explore short- and long-term strategies to encourage Chinese egg producers to adopt cage-free production systems, aligned with the intervention functions offered in the Behaviour Change Wheel.
